# Plant Carbonic Anhydrases: Structures, Locations, Evolution, and Physiological Roles

**DOI:** 10.1016/j.molp.2016.09.001

**Published:** 2017-01-09

**Authors:** Robert J. DiMario, Harmony Clayton, Ananya Mukherjee, Martha Ludwig, James V. Moroney

**Affiliations:** 1 Department of Biological Sciences, Louisiana State University, Baton Rouge, LA 70803, USA; 2 School of Chemistry and Biochemistry, University of Western Australia, Perth, WA 6009 Australia

**Keywords:** carbonic anhydrase, regulation, alternative splicing, physiological role

## Abstract

Carbonic anhydrases (CAs) are zinc metalloenzymes that catalyze the interconversion of CO_2_ and HCO_3_^−^ and are ubiquitous in nature. Higher plants contain three evolutionarily distinct CA families, αCAs, βCAs, and γCAs, where each family is represented by multiple isoforms in all species. Alternative splicing of CA transcripts appears common; consequently, the number of functional CA isoforms in a species may exceed the number of genes. CAs are expressed in numerous plant tissues and in different cellular locations. The most prevalent CAs are those in the chloroplast, cytosol, and mitochondria. This diversity in location is paralleled in the many physiological and biochemical roles that CAs play in plants. In this review, the number and types of CAs in C_3_, C_4_, and crassulacean acid metabolism (CAM) plants are considered, and the roles of the α and γCAs are briefly discussed. The remainder of the review focuses on plant βCAs and includes the identification of homologs between species using phylogenetic approaches, a consideration of the inter- and intracellular localization of the proteins, along with the evidence for alternative splice forms. Current understanding of βCA tissue-specific expression patterns and what controls them are reviewed, and the physiological roles for which βCAs have been implicated are presented.

## Teaser

Carbonic anhydrase catalyzes the interconversion of carbon dioxide and bicarbonate, the two forms of inorganic carbon used in multiple metabolic pathways in plants. This review covers the current understanding of the numerous and diverse carbonic anhydrase isoforms found in terrestrial plants. The regulation, alternative splicing, and physiological roles of the different carbonic anhydrase isoforms are discussed.

## Introduction

### Carbonic Anhydrases Are Essential for Photosynthetic Organisms and Their Intracellular Location Is Critical

Carbonic anhydrases (CAs) play essential roles in all photosynthetic organisms. In cyanobacteria, CAs located in the carboxysome are required for the conversion of accumulated HCO_3_^−^ to CO_2_ for fixation by ribulose 1,5-bisphosphate carboxylase/oxygenase (Rubisco) (Espie and Kimber, 2011). Loss of this carboxysomal CA results in abnormal growth of cyanobacteria when they are grown at ambient levels of CO_2_ (Price and Badger, 1989). In *Chlamydomonas reinhardtii*, a thylakoidal CA is necessary for the functioning of the CO_2_ concentrating mechanism (CCM) (Moroney and Ynalvez, 2007), and in the diatom, *Pheodactylum tricorutum*, a CA in the pyrenoid is required for the CCM of this species (Harada et al., 2005). In these organisms, the conversion of HCO_3_^−^ to CO_2_ for Rubisco is needed in a very specific location in the cell. In addition, the CCM of C_4_ plants requires CA activity specifically in the mesophyll (M) cell cytosol (Gutierrez et al., 1974). While the correct inter- and intracellular location of CAs is essential for efficient physiological functioning of photosynthetic organisms, it is also important that CA activity is not present in certain organelles or cell types. Price et al. (1992) demonstrated this when they transformed cyanobacteria with a gene encoding a human CA. This CA was expressed in the cytoplasm of the *Synechocystis* cells, effectively short circuiting the CCM (Price et al., 1992). Similarly, a defective C_4_ CCM resulted when a cytosolic CA was expressed in bundle-sheath (BS) cells of the C_4_ plant *Flaveria bidentis* (Ludwig et al., 1998). Recently, a number of research initiatives have been working to improve photosynthesis in plants by introducing CCM components from cyanobacteria, algae, or C_4_ plants into terrestrial C_3_ plants. While the introduction of active transporters and enzymes is required for these initiatives to work, it is also necessary to know where the endogenous CAs are active within the recipient plant, as introducing CA activity in the wrong location could short circuit attempts to improve photosynthesis.

This review focus on what is known about the genes encoding CA, and the locations of the CA isoforms in both C_3_ and C_4_ plants. Up-to-date research on the physiological roles of the different CA isoforms is also covered, as well as our current understanding of the molecular changes that were responsible for the evolution of the genes encoding C_4_-associated CAs from their ancestral C_3_ orthologs.

## Plants Have Three Types of Carbonic Anhydrases

**Figure 1:**
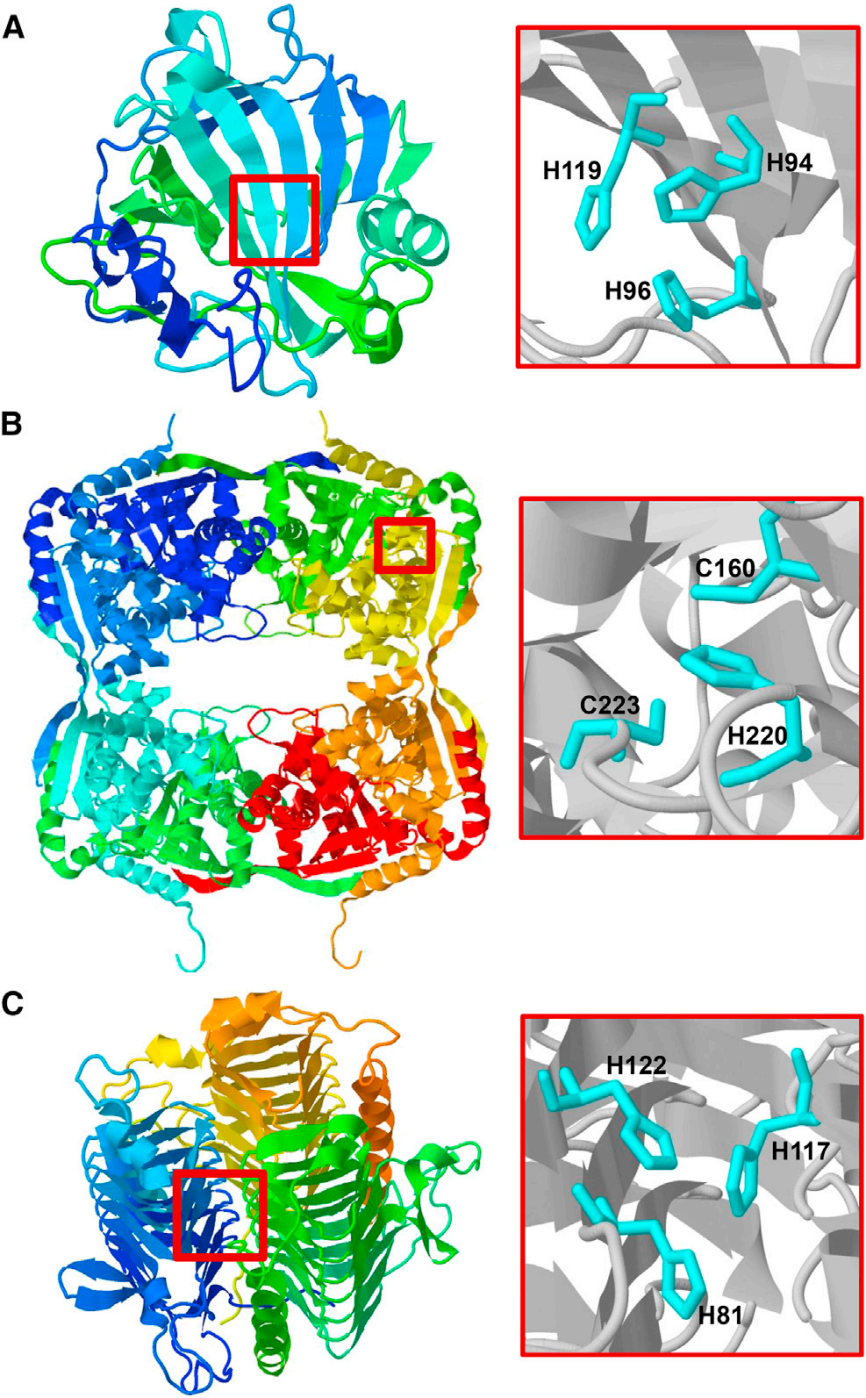
Structures of α, β, and γ Carbonic Anhydrase Proteins with Their Active Site Architecture. **(A)** The human CAII monomer (Mangani and Håkansson, 1992) mostly consists of β strands and contains a single active site with three zinc coordinating histidine residues. **(B)** The *Pisum sativum* βCA octamer (Kimber and Pai, 2000) contains eight active sites where each zinc is coordinated by two cysteines and a histidine. **(C)** The *Methanosarcina thermophila* γCA (Iverson et al., 2000) forms a trimer with three active sites. Although the γCA active site also contains three histidine resides, one monomer provides the H81 and H122 residues, while a second monomer provides the H117 residue to form the γCA active site. Red boxes indicate the enlarged locations of each protein structure to display their active-site architecture. CA protein structures and active-site images were generated using Jmol (http://www.jmol.org/).

All CAs are zinc metalloenzymes that catalyze the interconversion of CO_2_ and HCO_3_^−^. The enzymes are ubiquitous in nature and are an example of convergent evolution, as multiple, structurally and sequentially distinct families of CA have been discovered (Hewett-Emmett and Tashian, 1996). Plants have three types of CA: α-, β-, and γ-type CAs (Moroney et al., 2001). The α-type CA (αCA) was first found in erythrocytes and was the first CA family discovered (Brinkman et al., 1932, Meldrum and Roughton, 1932). The majority of the enzyme is composed of 10 β strands that create a large central β sheet, which is surrounded by seven α helices on the periphery of the protein (Figure 1A; Liljas et al., 1972). The zinc at the αCA active site is coordinated by three His residues and one water molecule organized in a tetrahedral conformation (Liljas et al., 1972, Eriksson et al., 1988, Håkansson et al., 1992), and is located in the central part of the protein, at the bottom of a cone-shaped crevice (Liljas et al., 1972). While most αCAs are monomers, multimeric αCAs have been discovered as well as αCAs containing extra domains (Ishida et al., 1993, Hilvo et al., 2008). However, even in multimeric αCAs, the zinc ion is always coordinated by His residues from a single polypeptide.

The β-type CA (βCA) was first discovered in plants (Burnell et al., 1990, Fawcett et al., 1990, Roeske and Ogren, 1990), and its protein sequence and structure are very different from that of the αCAs. In βCAs, the zinc ion is coordinated by two Cys residues, one His residue, and a water molecule (Figure 1B; Kimber and Pai, 2000). The structure of a βCA monomer is mostly composed of α helices that surround a β sheet consisting of four parallel β strands. There is also a fifth, C-terminal β strand involved in the oligomerization of βCA (Kimber and Pai, 2000). The functional unit of the βCA is a dimer, although the most common βCA oligomerization is a tetramer (Kimber and Pai, 2000, Rowlett, 2010). The βCA dimer is formed via extensive interactions created by two N-terminal α helices of one monomer wrapping around the second monomer and by minor hydrogen bonding between the second β strand of each monomer (Kimber and Pai, 2000). Tetramers are formed by interactions made primarily by the fifth, C-terminal β strand (Kimber and Pai, 2000). In pea, the chloroplastic βCA forms an octamer. For some βCAs, dicots have a unique C-terminal extension of the fifth β strand, whereas monocots do not (Kimber and Pai, 2000, Rowlett, 2010). Octamers are formed via slightly different interactions with these fifth β-strand extensions (Kimber and Pai, 2000, Rowlett, 2010).

The γ-type CA (γCA) was first discovered in archaea (Alber and Ferry, 1994) but has since been found in photosynthetic bacteria (Price et al., 1993, Peña et al., 2010) and in plants (Parisi et al., 2004). The first crystal structure of γCA from *Methanosarcina thermophila* was reported by Kisker and colleagues in 1996 (Figure 1C). Much like the active site of αCA, the active site of γCA also contains a zinc atom coordinated by three His and a water molecule (Kisker et al., 1996). However, unlike the structure of αCAs, which are monomers, the functional unit of γCA is a trimer, with three active sites spanning the monomer-monomer interfaces. The zinc ion is coordinated by His residues provided by two different subunits (Kisker et al., 1996). A β-strand region dominates the structure of γCA and consists of seven complete turns creating a left-handed β helix (Kisker et al., 1996). Each full turn contains three β strands making the β helix look like an equilateral triangle from the top view (Kisker et al., 1996). In photosynthetic organisms, γCA may contain extra domains as seen in the cyanobacterial CcmM proteins of cyanobacteria that have two or three repeated C-terminal domains with high similarity to the small subunit of Rubisco (Long et al., 2007). In cyanobacteria, CcmM sometimes functions as an active CA, but some CcmM proteins do not have activity (Peña et al., 2010, de Araujo et al., 2014). However, CcmM is thought to organize the packing of Rubisco in the carboxysome even when it does not have CA activity.

## Plants Have Multiple Genes Encoding All Three Types of Carbonic Anhydrases

Plants have a large number of genes encoding CA. There are 17 distinct genes in total encoding all α, β, and γ, isoforms in *Arabidopsis*, including two γ-like CAs (Table 1). A similar number of genes are present in the genomes of other plant species, including mosses, monocots, and dicots (Table 1). Since plants can be polyploids or paleopolyploids, having undergone genome duplication in the past, the number of CA genes may be much higher. For example, in soybean, a diploid plant thought to have undergone genome duplication relatively recently, the total number of genes coding for CA is in excess of 25. Genes encoding CA are expressed in almost all tissues of the plant and CA isoforms can be found in most intracellular compartments, such as chloroplasts, mitochondria, the plasma membrane, and the cytoplasm.

## Green Algae Also Contain the Three Types of Carbonic Anhydrase

It is likely that the numbers and types of CA are quite ancient in the plant lineage as the unicellular green alga *Chlamydomonas reinhardtii* also has multiple genes encoding α-, β- and γCAs. *C. reinhardtii* has three genes encoding αCA, six encoding βCA, and three encoding γCA and γCA-like proteins (Mitra et al., 2005). The CA isoforms of *C. reinhardtii* are found throughout the algal cell: in the periplasmic space (cell wall), chloroplast, cytoplasm, and mitochondria. As seen in higher plants, the *C. reinhardtii* γCAs and γCA-like proteins are mitochondrial with evidence suggesting they are part of Complex I of the mitochondrial electron transport chain, and the βCAs are found in similar intracellular locations as in higher plants, with isoforms in the mitochondria, chloroplast, and cytoplasm. However, two of the *C. reinhardtii* βCAs have hydrophobic C-terminal extensions (Ynalvez et al., 2008), this is not observed so far in terrestrial plants. In addition, in *C. reinhardtii* the αCAs seem to play different physiological roles. In terrestrial plant species, only β- and γCAs have been implicated in CCMs, whereas two αCAs play important roles in the CCM of *C. reinhardtii*: CAH1, which is located in the periplasmic space, and CAH3, which is found in the thylakoid lumen. For more information on *Chlamydomonas* CAs, see Moroney et al. (2011).

## Plant α Carbonic Anhydrases

**Figure 2:**
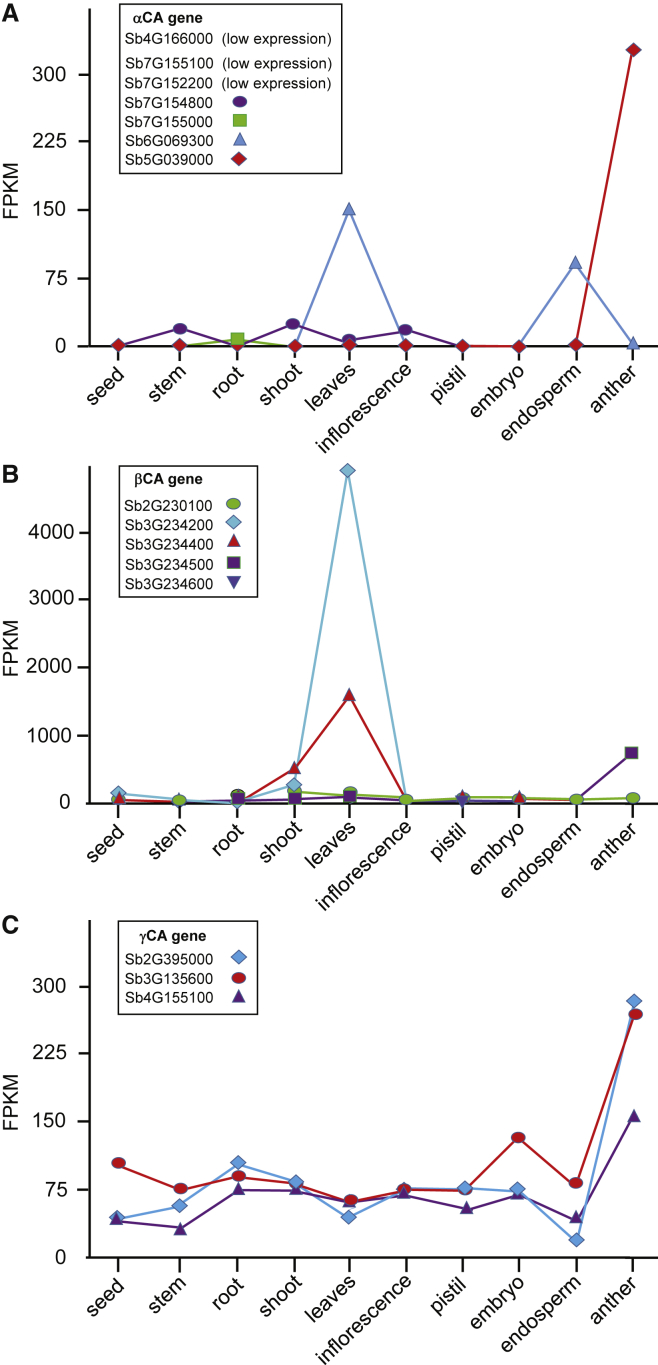
Relative Expression of Carbonic Anhydrases in *Sorghum bicolor* Organs. **(A)** α, **(B)** β, and **(C)** γ carbonic anhydrase expression in different organs of *Sorghum bicolour* taken from MOROKOSHI - The Sorghum Transcriptome Database (Makita et al., 2015), in fragments per kilobase of transcript per million mapped reads (FPKM). Note that the y axis for the βCA expression is different than that of the αCA or γCA graphs.

αCAs are the largest CA gene family in most plants, but they are also the least studied. The scarcity of published work on αCAs is most likely because the proteins are not highly abundant in leaves and roots. In *Arabidopsis*, complete expressed sequence tags (ESTs) exist for only three of the eight *αCA* genes, and RNA-seq data also only poorly cover the other five annotated genes. *Arabidopsis αCA8* is clearly a pseudogene as it encodes in-frame stop codons. Limited expression information for some *αCA* genes is available through RNA-seq data on genome sites. Interestingly, in sorghum, the αCA *Sb5G039000* is expressed specifically in anthers (Figure 2; Makita et al., 2015), while in *Medicago trunculata*, the αCAs *Mt1g059900* and *Mt1g059940* are expressed in root nodules (Tang et al., 2014). In *Arabidopsis*, *αCA2* is expressed in trichomes of the leaf. Clearly at least some *αCA* genes show quite specific organ or tissue expression patterns. To date, there are no reports of plants where one or more *αCA* genes have been disrupted.

There is very little information on the intracellular location of αCA isoforms. Two reports suggest that *Arabidopsis* αCA1 is a chloroplastic protein (Villarejo et al., 2005, Blanco-Rivero et al., 2012). The targeting of this αCA1 is unusual as it moves through the endoplasmic reticulum and is glycosylated. However, the location of αCA1 in the chloroplast has not yet been confirmed by proteomic studies. Possibly the amount of αCA in leaves is low, or perhaps the glycosylation obscures its detection. There are no reports on the subcellular location or function of αCAs from other plants at this time.

## Plant γ Carbonic Anhydrases

Genes encoding γCAs and γCA-like proteins have been found in all plants. In fact, every species appears to have at least two genes encoding γCAs and at least one encoding a γCA-like protein (Table 1). For example, *Arabidopsis* has three γ*CA* genes and two genes encoding γCA-like proteins (Parisi et al., 2004, Perales et al., 2004). γCAs are well conserved in photosynthetic organisms, from green algae, to mosses, monocots, and dicots. While no higher plant γCA with CA activity has been identified, the proteins have the active-site residues found in γCAs from archaebacteria and cyanobacteria. In contrast, the γCA-like proteins do not have the required Zn coordinating amino acid residues. While γCAs are encoded by the nucleus, they are mitochondrial proteins. They have been shown to be part of the mitochondrial Complex I (NADH-ubiquinone oxidoreductase), and make up an extrinsic domain known as the carbonic anhydrase domain of the oxidoreductase (Sunderhaus et al., 2006), which is composed of three subunits: two γCA subunits and one γCA-like subunit (Klodmann et al., 2010). The γCA and γCA-like proteins are part of nine plant-lineage-specific subunits.

The expression level of genes coding for γCA and γCA-like isoforms is average or above in almost all tissues for which expression data are available (Figure 2). This is not surprising for a subunit of Complex I as the mitochondrial electron transport chain is found in most plant tissues and cell types. If either *AtγCA1* or *AtγCA2* is knocked out, there is a small reduction in Complex I (Perales et al., 2005); however, if both *AtγCA1* and *AtγCA2* are knocked out, the plant is profoundly and adversely affected. The *γca1γca2* mutants lack Complex I altogether, and do not produce viable seed, having to be maintained using an embryo rescue method, which involves supplying the embryos with sucrose in the growth medium (Fromm et al., 2016a, Fromm et al., 2016b). The double mutants also exhibit high levels of Complexes II and IV (succinate dehydrogenase and cytochrome oxidase, respectively), and the alternative oxidase, and in contrast, reduced levels of photosynthetic proteins (Fromm et al., 2016c).

## Plant β Carbonic Anhydrases

**Figure 3:**
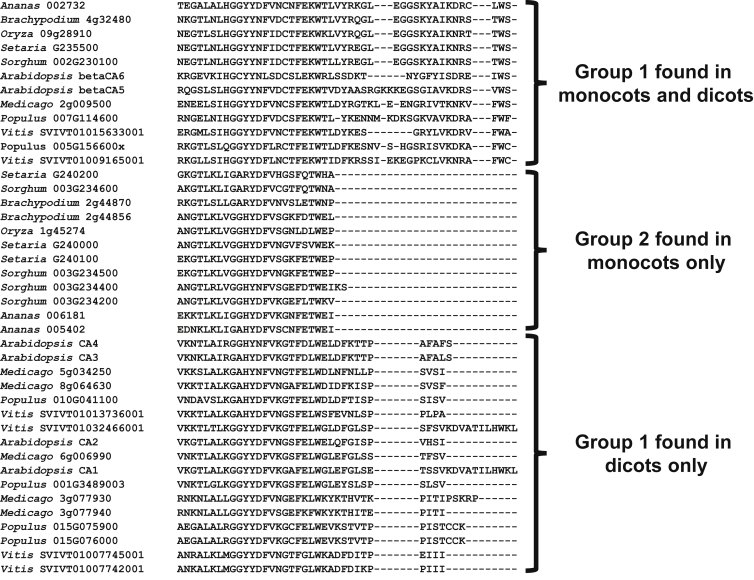
Multiple Sequence Alignment of C-termini of β Carbonic Anhydrase Proteins from Different Plants. Sequences were aligned using Clustal Omega (http://www.ebi.ac.uk/Tools/msa/clustalo/; Sievers et al., 2011). Sequences for *Physcomitrella patens* (Lang et al., 2005*;*Rensing et al., 2005, Zimmer et al., 2013), *Selaginella moellendorffii* (Banks et al., 2011), *Medicago truncatula* (Young et al., 2011, Tang et al., 2014), *Vitis vinifera* (Jaillon et al., 2007), *Populus trichocarpa* (Tuskan et al., 2006, Du et al., 2015, Ye and Zhong, 2015), *Brachypodium distachyon* (Vogel et al., 2010), *Oryza sativa* (Ouyang et al., 2007), *Setaria italic* (Bennetzen et al., 2012), *Sorghum bicolor* (Makita et al., 2015) were obtained from Phytozome (https://phytozome.jgi.doe.gov). Sequences for *Arabidopsis thaliana* were obtained from TAIR (Lamesch et al., 2011). Sequences for *Ananas comosus* were obtained from CoGe (https://genomevolution.org; Ming et al., 2015).

Plants have a moderate number of *βCA* genes, usually between four and seven (Table 1). There have been a number of studies on the βCAs as they are highly expressed in leaf tissue (Figure 2). βCAs have been found in chloroplasts, mitochondria, the cytosol, and the plasma membrane of *Arabidopsis* (Table 2), and in the cytosol and chloroplasts of many plants. When the predicted amino acid sequences of βCAs from monocots and dicots are aligned, the isoforms can be divided roughly into three groups (Figure 3). One group was found in all monocots and dicots considered (Figure 3) and is represented by *Atβ*CA5 and *Atβ*CA6, which localize to the chloroplast and mitochondria, respectively. The second group was found only in dicots, and the *Arabidopsis Atβ*CA1, *Atβ*CA2, *Atβ*CA3, and *Atβ*CA4 proteins are in this group. All dicots examined had at least two βCA proteins in this group (Figure 3). The third group contains only monocot CAs, and these proteins are known to localize to the chloroplast and cytosol. The length of the C termini of the proteins was an indicator for the group in which an isoform clusters. The monocot-specific proteins were the shortest, with the dicot-specific isoforms about 10 amino acids longer and the *Atβ*CA5/*Atβ*CA6-related proteins, found in all species examined, about 20 amino acids longer (Figure 3).

As the βCAs have been the most intensely studied family of plant CAs, the remainder of this review focuses on this group.

## Evidence of Alternative Splicing of β Carbonic Anhydrase Transcripts

**Figure 4:**
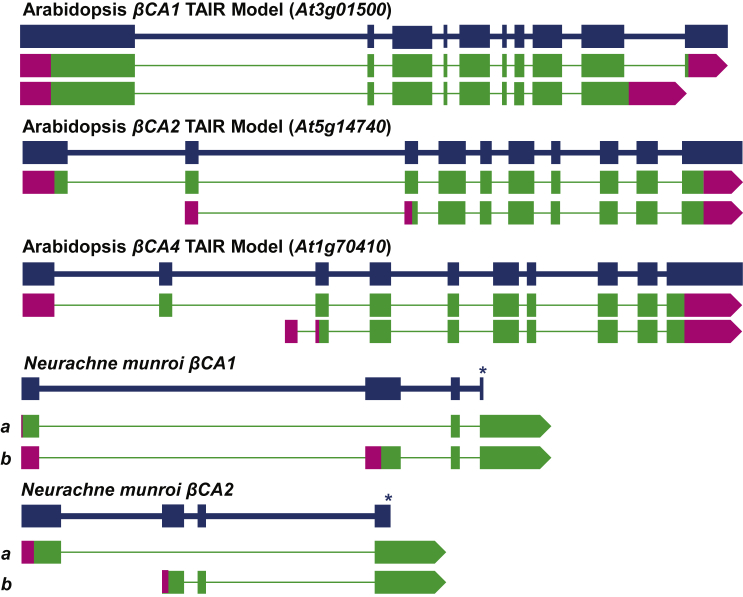
Alternative Splicing of β Carbonic Anhydrase Genes in *Arabidopsis thaliana* and *Neurachne munroi*. Blue lines indicate genomic DNA with larger boxes representing exons. Green and magenta boxes indicate exons present in different splice forms, with green representing open reading frame sequence and magenta representing untranslated regions. Asterisks indicate that the *Neurachne munroi CA1* and *CA2* genomic DNA sequences are incomplete; for each gene, exon 4 and the downstream exons are present in both splice forms, as represented by the green arrows. Data from DiMario et al. (2016) and Clayton et al. (2016).

Alternative splicing can result in a single gene coding for multiple proteins that may show tissue-specific expression patterns and/or be targeted to different organelles of the cell. Deposited ESTs in TAIR, as well as RNA-seq data (Oh et al., 2014), indicate that transcription of the *AtβCA1* gene may result in two different mRNAs (Figure 4). The RNA variants arise from the splicing of the ninth and tenth exons, where one variant has all 10 exons, and the other has an extended ninth exon, making proteins that differ slightly at their C termini. Two different transcription start sites for *AtβCA2* (Figure 4) are predicted to encode two *Atβ*CA2 isoforms with different N termini, resulting in the two proteins having different projected destinations in the plant cell. *AtβCA4* is another example of a gene that can produce multiple mRNA forms (Figure 4; Aubry et al., 2014), due to different transcription start sites (Figure 4). The shorter *AtβCA4* mRNA lacks the first two exons, encoding a different N terminus relative to the longer form. Interestingly, RNA-seq data for *AtβCA4* indicate that the shorter mRNA has a unique first exon that is not present in the longer transcript and is expressed in both roots and leaves (DiMario et al., 2016). In contrast, the longer *AtβCA4* mRNA is expressed specifically in leaves of *Arabidopsis* (DiMario et al., 2016).

In *Neurachne munroi* leaves, four *βCA* transcripts are expressed that are derived from two genes by way of alternative splicing (Figure 4; Clayton et al., 2016). For each gene, the alternatively spliced transcripts encode polypeptides that share the same active-site residues but have distinct N termini, thereby influencing the subcellular location of each isoform (Clayton et al., 2016). In *N. munroi*, the *CA1* gene encodes *Nm*CA1a and *Nm*CA1b. Exon 2 is not present in *NmCA1a* transcripts, such that in *CA1a* and *CA1b*, the initiating AUG and N-terminal regions are encoded in different frames (Figure 4; Clayton et al., 2016). In the case of the *NmCA2* gene, *NmCA2a* transcripts do not contain exons 2 and 3, while exon 1 is not present in the *NmCA2b* transcript, again resulting in each splice form encoding a distinct N-terminal region (Figure 4; Clayton et al., 2016). The same gene-transcript relationship was observed in all other *Neurachne* species for which transcript and genomic DNA sequences have been obtained (data not shown). While experimental evidence for alternative splicing of other monocot βCA genes has not been reported, parsing of EST databases and comparison with genome sequences suggest that alternative splice forms do exist in other species.

## Subcellular Locations of β Carbonic Anhydrase from Experimental Data

Linking CA to photosynthesis has been a major focus in the plant CA field. Studies using reverse transcription (RT)–PCR, microarrays, and RNA-seq have shown that all six *βCA* genes are expressed in leaves of *Arabidopsis* (Schmid et al., 2005, Fabre et al., 2007, Winter et al., 2007, Ferreira et al., 2008, Hu et al., 2010, Wang et al., 2014, DiMario et al., 2016); however, subcellular localization studies using green fluorescent protein (GFP) fusion constructs showed that not all of the proteins localize to the chloroplast. *Atβ*CA1 was shown to have a long chloroplast transit peptide over 100 amino acid residues in length (Fett and Coleman, 1994, Kim et al., 1994), and was later confirmed to be located in the chloroplast via GFP studies (Table 2; Fabre et al., 2007, Hu et al., 2015). *Atβ*CA2 and *Atβ*CA3 are cytosolic βCAs (Table 2; Fabre et al., 2007, DiMario et al., 2016) although *Atβ*CA2 is expressed at a much higher level than *Atβ*CA3 in *Arabidopsis* leaves (Schmid et al., 2005, Winter et al., 2007, Ferreira et al., 2008, Hu et al., 2010, DiMario et al., 2016). The long form of *Atβ*CA4, *Atβ*CA4.1, localizes to the plasma membrane while the short form, *Atβ*CA4.2, is cytosolic, as it no longer has a secretory transit peptide (Table 2; Fabre et al., 2007, DiMario et al., 2016). Studies using GFP fusions also showed that *Atβ*CA5 localizes to the chloroplast (Fabre et al., 2007), and *Atβ*CA6 is a mitochondrial form of the enzyme (Table 2; Fabre et al., 2007, Jiang et al., 2014).

To date, three transcripts encoding distinct βCA isoforms (CA1, CA2, and CA3) have been found in leaves of *Flaveria* species (Tetu et al., 2007, Tanz et al., 2009). Chloroplast import assays showed in both *F. bidentis* (a C_4_ species) and *F. pringlei* (a C_3_ species) that CA1 was imported into chloroplasts, while CA2 was not (Table 2; Tetu et al., 2007, Tanz et al., 2009). However, the subcellular location of CA3 was not conserved between the two species; *Fp*CA3 was found to be chloroplast targeted, while *Fb*CA3 was not (Table 2; Tetu et al., 2007, Tanz et al., 2009). Comparison of the predicted amino acid sequences of the CA3 polypeptides revealed that *Fb*CA3 lacks 71 amino acids at the N terminus, including the chloroplast transit peptide, when compared with *Fp*CA3, a situation that was proposed to be important to the molecular evolution of C_4_ photosynthesis in *F. bidentis* (Tanz et al., 2009).

The only monocot species so far in which the subcellular location of βCA isoforms has been experimentally determined are from the genus *Neurachne* (Clayton et al., 2016). GFP fusion constructs indicated that *N. munroi* CA2b is imported into tobacco chloroplasts or mitochondria, while *Nm*CA1a, *Nm*CA1b, and *Nm*CA2a localize to the cytosol (Table 2; Clayton et al., 2016). As in *Flaveria*, the location of the isoforms is not conserved between C_3_ and C_4_ *Neurachne* species. While the GFP localization experiments showed that *Na*CA1b from the C_3_ species *N. alopecuroidea* is cytosolic as predicted, *Na*CA1a is imported into the chloroplasts, in contrast with the cytosolic location of CA1a from *N. munroi* (Table 2; Clayton et al., 2016). Analyses of the predicted proteins indicated that 11 amino acids present in the N-terminal region of the *Na*CA1a polypeptide, but absent in *Nm*CA1a, is important for chloroplast targeting (Clayton et al., 2016).

## Predicting β Carbonic Anhydrase Subcellular Locations

Numerous algorithms exist for predicting protein subcellular location. Predicted βCA amino acid sequences from various monocot and dicot species were analyzed using four algorithms: Predotar, ChloroP, TargetP, and MultiLoc (Supplemental Table 1; Emanuelsson et al., 1999, Small et al., 2004, Höglund et al., 2006, Emanuelsson et al., 2007). The results indicate that each species contains at least one isoform that is chloroplast targeted and at least one isoform that is likely cytosolic, and that many also contain a mitochondrial βCA (Supplemental Table 1). Comparison with actual experimental results for *Arabidopsis*, *Flaveria*, and *Neurachne* (Table 2) indicates that these predictions are often but not always correct (cf. Table 2 and Supplemental Table 1).

The above results indicate that all predictions must be experimentally tested, keeping in mind that the proteins resulting from different splice forms of a single gene may have different subcellular locations (Table 2; Clayton et al., 2016, DiMario et al., 2016). In addition, predicting βCA subcellular location based on the products of orthologous genes in closely related species may not assist in assigning location since it has been shown that locations are not conserved between closely related C_3_ and C_4_ species (Tanz et al., 2009, Clayton et al., 2016).

## Organ-, Tissue-, and Cell-Type-Specific Expression of β Carbonic Anhydrases

To date, the specific expression patterns of all identified βCA isoforms have been reported in the scientific literature for only three species: *Arabidopsis thaliana* (Schmid et al., 2005, Fabre et al., 2007, Winter et al., 2007, Ferreira et al., 2008, Wang et al., 2014, DiMario et al., 2016), *F. bidentis* (Tetu et al., 2007), and *F. pringlei* (Tanz et al., 2009). An in-depth CA expression study of *Arabidopsis* rosette leaves found that *AtβCA1*, *AtβCA2*, and *AtβCA4* are the most highly expressed CA genes in M cells, and *AtβCA1*, *AtβCA4*, and *AtβCA6* are the most highly expressed CA genes in guard cells (Hu et al., 2010). Many studies also report CA gene expression in *Arabidopsis* roots (Schmid et al., 2005, Fabre et al., 2007, Winter et al., 2007, Wang et al., 2014, DiMario et al., 2016), with RNA-seq data showing that *AtβCA2* and *AtβCA3* are the two lowest expressed βCA genes in *Arabidopsis* roots, whereas *AtβCA4* and *AtβCA5* are the two most highly expressed genes (DiMario et al., 2016). Microarray analyses indicated all *AtβCA* genes are expressed in roots of *Arabidopsis*, albeit *AtβCA1*, *AtβCA2*, and *AtβCA3* are expressed at very low levels (Schmid et al., 2005, Winter et al., 2007). Results of an RT–PCR analysis showed that transcripts from most of the *AtβCA*s genes are found in *Arabidopsis* roots with the exception of *AtβCA2* (Wang et al., 2014), whereas another RT–PCR experiment found that *AtβCA3* and *AtβCA6* show the highest expression in roots (Fabre et al., 2007). Both RT–PCR studies found that *Arabidopsis* genes encoding βCA are expressed in stems and floral tissues (Fabre et al., 2007, Wang et al., 2014), and all six *AtβCA* genes are expressed in *Arabidopsis* stem and floral tissues according to microarray analyses (Schmid et al., 2005, Winter et al., 2007). Interestingly, the microarray data indicate that all six *AtβCA* genes are expressed in *Arabidopsis* seeds although, with the exception of *AtβCA5* and *AtβCA6*, their expression diminishes as the seeds develop (Schmid et al., 2005, Winter et al., 2007).

In *F. pringlei*, RT–quantitative (q)PCR assays indicated that transcripts encoding *Fp*CA1 and *Fp*CA3 were primarily expressed in leaves, whereas *FpCA2* was expressed in leaves, roots, and flowers (Tanz et al., 2009). By contrast, transcripts encoding CA1, CA2, and CA3 were detected in *F. bidentis* leaves, roots, and flowers, but *FbCA1* and *FbCA3* transcripts were most abundant in leaves, whereas *FbCA2* mRNA levels were consistent among the three tissues (Tetu et al., 2007).

While numerous reports have shown that the majority of CA activity in leaves of C_4_ species is in the M cells (Gutierrez et al., 1974, Ku and Edwards, 1975, Burnell and Hatch, 1988), the location of multiple βCA isoforms within a leaf has not been comprehensively examined in any species. In *F. bidentis*, immunocytochemical experiments showed that CA is expressed predominantly in M cells and is undetectable in BS cells (Tetu et al., 2007). Presumably this is the *Fb*CA3isoform previously shown to be essential for C_4_ photosynthesis (von Caemmerer et al., 2004). The tissue/cell type-specific expression patterns of *Fb*CA1 and *Fb*CA2 were not examined.

Recently, advances in laser-capture microdissection and next-generation sequencing have enabled M and BS cell transcriptomes to be obtained. Comparative transcriptome analyses of leaf M and BS cells of *Panicum virgatum* (Rao et al., 2016), *Setaria viridis* (John et al., 2014), *Gynandropsis gynandra* (formerly *Cleome gynandra*; Aubry et al., 2014), and *Zea mays* (Li et al., 2010, Chang et al., 2012) showed that particular *βCA* transcripts are enriched in M cells. Specifically, *Pavir.J08788* and *Pavir.J05107* transcripts are approximately three times more abundant in M cells than in BS cells in *P. virgatum* (Rao et al., 2016), and a similar fold difference is also observed for two transcripts encoding βCA in *G. gynandra*, *GgβCA1* and *GgβCA2* (*At3g01500* and *At5g14740* orthologs; Aubry et al., 2014), one transcript in *S. viridis* (*Si03061m.g* ortholog; John et al., 2014), and one in *Z. mays* (*GRMZM2G414528*; Li et al., 2010, Chang et al., 2012). However, in the latter two species, there are several *βCA* transcripts that show much higher M-specific abundance, with at least a 20-fold enrichment compared with BS cells. These include *Si003885m.g* in *S. viridis* (John et al., 2014), and *GRMZM2G121878*, *GRMZM2G348512*, and *GRMZM2G094165* in *Z. mays* (Li et al., 2010, Chang et al., 2012, Rao et al., 2016). Interestingly, the maize *GRMZM2G145101* transcript is the only *βCA* transcript identified so far that shows higher abundance in BS cells than M cells (Li et al., 2010, Chang et al., 2012, Rao et al., 2016), while no *βCA* mRNAs from *S. viridis*, *G. gynandra*, and *P. virgatum* show this pattern. Taken together, the current findings suggest that within a C_4_ species, genes encoding the different βCA isoforms show different tissue- and cell-specific expression patterns, with some isoforms showing preferential expression in the leaf M cells.

## Activity of β Carbonic Anhydrase in C_3_ and C_4_ Species

Total leaf CA activity within herbaceous dicotyledonous plants ranges from 2- to 10-fold (Everson and Slack, 1968, Atkins et al., 1972, Triolo et al., 1974, Reed and Graham, 1981, Hatch and Burnell, 1990, Gillon and Yakir, 2001, supplementary material), whereas the leaves of some monocotyledons reportedly contain 1000 times more CA activity than other monocot species (Everson and Slack, 1968, Atkins et al., 1972, Triolo et al., 1974, Reed and Graham, 1981, Burnell and Hatch, 1988, Hatch and Burnell, 1990, Gillon and Yakir, 2001, supplementary material; Cousins et al., 2008). Total leaf CA activity in C_3_ monocots can be 500 times higher than that of C_4_ monocots (Everson and Slack, 1968, Triolo et al., 1974, Reed and Graham, 1981, Hatch and Burnell, 1990, Gillon and Yakir, 2001, supplementary material), while leaves of herbaceous C_4_ dicots demonstrate total CA activities that fall within the range of values for C_3_ dicot leaves (Everson and Slack, 1968, Atkins et al., 1972, Reed and Graham, 1981, Hatch and Burnell, 1990, Gillon and Yakir, 2001, supplementary material).

Very few studies have looked at CA activity in both isolated M and BS cells from C_4_ plant leaves; however, depending on the comparison being made, this is important. As it is the cytosolic CA in M cells that is associated with the C_4_ CCM, total leaf CA activity measurements may be misleading. Two forms of CA were isolated from *Amaranthus cruentus* leaves (Guliev et al., 2003). One form was found associated with the chloroplasts of the BS cells and was responsible for 8% of total leaf CA activity. In contrast, the other form was found in the M cell cytoplasmic fraction, where it represented 62% of the total CA activity in amaranth leaves (Guliev et al., 2003). In another C_4_ dicot, *Flaveria bidentis*, BS cell CA activity was found to contribute 0.5% of total leaf CA activity (Ludwig et al., 1998).

Burnell and Hatch (1988) also found low CA activity in BS cells of species representing the three C_4_ subtypes: NADP-malic enzyme (NADP-ME), NAD-malic enzyme (NAD-ME), and phospho*enol*pyruvate carboxykinase (PCK). In the two NADP-ME-subtype species examined, sorghum and maize, BS cell CA activity was 1.8% and 1.6%, respectively, of total leaf CA activity. The activity of CA in the BS of NAD-ME-type (*P. miliaceum* and *Atriplex spongiosa*) and PCK-type species (*Urochloa panicoides* and *Chloris gayana*) was even lower, representing just 0.5%–0.8% of total leaf CA activity (Burnell and Hatch, 1988). These results suggested low CA activity in the BS was a requisite for efficient functioning of the C_4_ pathway (Burnell and Hatch, 1988). This idea was later supported by a transgenic approach in which wild-type (WT) plants of the C_4_ species *F. bidentis* were transformed with the sequence encoding mature tobacco CA (i.e., no chloroplast transit peptide) that was under the control of a constitutive promoter (Ludwig et al., 1998). This allowed tobacco CA expression in the cytosol of all cells, including leaf BS cells. The transformants showed increased BS leakiness to inorganic carbon (C_i_), reduced rates of photosynthesis, and an impaired CCM (Ludwig et al., 1998). Together these results support the idea that in C_4_ plants demonstrating Kranz leaf anatomy, the strict M and BS cell compartmentalization of CA is essential for the proper functioning of the C_4_ CCM.

## Regulation of Cell-Type-Specific Expression of β Carbonic Anhydrase in C_4_ Plants

Progress has been made in our understanding of *cis* elements and chromatin marks that control the preferential accumulation of transcripts encoding βCA in M cells of C_4_ species; however, the associated *trans*-acting factors remain elusive. Studies suggest several regulatory mechanisms were already present in ancestral C_3_ genes coding for βCA but were modified through recruitment of posttranscriptional pathways or the binding of different transcription factors during the evolution of C_4_ photosynthesis.

In leaves of the C_4_ species *Gynandropsis gynandra*, transcripts encoding the homolog of the *Arabidopsis* plasma-membrane-associated CA (*Atβ*CA4; Fabre et al., 2007, Hu et al., 2010, Kajala et al., 2012), showed abundances similar to the levels of mRNAs coding for other C_4_-associated proteins (Bräutigam et al., 2011, Kajala et al., 2012). Elements in either the 5′-untranslated region (UTR) or 3′-UTR of the *G. gynandra* gene encoding βCA4 were found to be sufficient for M-cell-specific expression using GUS fusion constructs (Kajala et al., 2012). Similar sequences in the 5′- and 3′-UTRs of *AtβCA4* were also shown to independently direct M-cell-specific expression when they were used to transform *G. gynandra*.

A more recent study showed the 3′-UTR of a second *G. gynandra* CA gene, *GgCA2*, for which high transcript levels are found in leaves, and the homologous region from *AtβCA2* also direct preferential accumulation of GUS in M cells (Williams et al., 2016). A common, nine-nucleotide motif in these *CA2* 3′-UTRs, as well as in the 5′- and 3′-UTRs of *AtβCA4* and *GgCA4*, were identified as sufficient to direct M cell-specific expression and was designated MEM2 for mesophyll expression module 2 (Williams et al., 2016). This study also showed that the MEM2 element does not control the level of *GgCA4* gene expression but instead works post-transcriptionally through a mechanism that increases the amount of CA4 protein made in M cells relative to the BS. The high levels of *GgCA4* transcripts in *G. gynandra* M cells appear to result from the loss of elements in the promoter region and introns from the ancestral *CA4* gene that repress its expression in C_3_ species (Williams et al., 2016).

Epigenetic marks have been identified that contribute to M cell-specific expression of genes encoding CA isoforms important in C_4_ photosynthesis. Trimethylation of the Lys residue at position 4 on histone H3 (H3K4me3) is associated with transcriptionally active genes and is enriched in the 5′-region of the transcribed sequence (Santos-Rosa et al., 2002). Heimann et al. (2013) found the gene encoding one of the C_4_-associated CAs in maize, GRMZM2G121878, showed a high ratio of H3K4me3 to the dimethylated form (H3K4me2) in M cells. This is consistent with the methylation state of histone H3K4 found at analogous positions in genes coding for the C_4_-associated forms of PEPC and pyruvate phosphodikinase (PPDK), which also show preferential accumulation of transcripts in M cells (Heimann et al., 2013).

## Physiological Roles of β Carbonic Anhydrases

The total number of genes encoding CA is similar in dicots and monocots, and in plants using C_3_ and C_4_ photosynthesis (Table 1; Williams et al., 2012), with some of these genes encoding isoforms that likely perform the same function in all species. However, as highlighted above, differences in total CA activity and control of βCA expression patterns have been detected within and between these plant groups, and are responsible for the very specific physiological roles exhibited by some of the enzymes.

### C_3_ Photosynthesis

**Figure 5:**
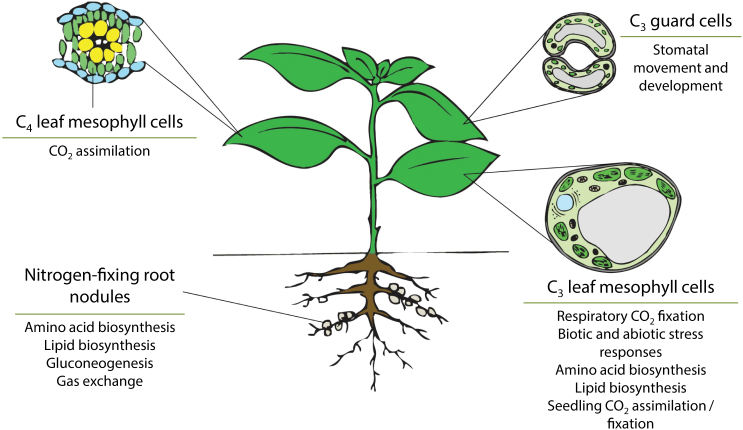
Schema Illustrating the Physiological Functions of β Carbonic Anhydrases in Plant Cells and Organs. In leaf mesophyll cells of C_4_ plants, a cytosolic βCA catalyzes the first step in C_4_ photosynthesis. βCAs are involved in a CO_2_ sensing pathway in guard cells and implicated in stomatal development. A number of roles have been attributed to βCAs found in leaf mesophyll cells of C_3_ plants, including involvement in refixation of respiratory CO_2_, stress responses, amino acid and lipid biosynthesis, and seedling establishment. In nitrogen-fixing root nodules of legumes, βCAs are implicated in different functions during nodule maturation, including roles in primary metabolism and gas exchange. Note: the roles of βCA in C_3_ guard cells and C_3_ leaf mesophyll cells are likely to be performed by homologs in the corresponding cell types of C_4_ plants. See text for details and references.

In the leaves of C_3_ plants, the majority of CA activity localizes to M cell chloroplasts (Everson and Slack, 1968, Everson, 1970, Poincelot, 1972), where the enzyme can make up 1%–2% of total leaf protein (Okabe et al., 1984, Peltier et al., 2006). Although a major component of the C_3_ leaf proteome, the actual role of βCAs in C_3_ photosynthesis remains ambiguous (Figure 5). Initial suggestions included the conversion of HCO_3_^−^ to CO_2_ to ensure maximum rates of fixation by Rubisco (Everson, 1970, Poincelot, 1972, Werdan and Heldt, 1972), facilitating the diffusion of CO_2_ across the chloroplast membranes (Poincelot, 1972), buffering short-term changes in pH in the chloroplast stroma induced by changing light conditions (Jacobson et al., 1975), and the hydration of compounds other than CO_2_ (Jacobson et al., 1975). However, to date, none of these proposed functions has strong empirical support.

To determine the physiological function of C_3_ plant chloroplast βCA, WT tobacco plants were transformed with antisense constructs directed against transcripts encoding the tobacco chloroplastic βCA isoform (Majeau et al., 1994, Price et al., 1994). Primary transformants with 1%–2% of the CA activity of WT plants showed no significant differences in CO_2_ assimilation rates, Rubisco activity, and chlorophyll content relative to WT plants.

More recently, *Arabidopsis* antisense transformants and knockout lines of *βCA1* were examined (Ferreira et al., 2008), and these plants did demonstrate an obvious phenotype. Both lines of transformants showed reduced seedling survival that could be rescued by including sucrose in the growth medium, or growing the seedlings in elevated CO_2_. The cotyledons were found to have compromised CO_2_ assimilation rates that resulted in the observed reduced seedling establishment before development of the first true leaves (Figure 5). However, when the transformants did survive, the mature plants showed no phenotypic differences from WT plants, strongly suggesting that *Atβ*CA1 plays no direct role in photosynthesis of mature *Arabidopsis* plants (Ferreira et al., 2008).

### C_4_ Photosynthesis

In contrast to C_3_ plants, most βCA activity in C_4_ plants is found in the cytosol of M cells (Gutierrez et al., 1974), where it catalyzes the first reaction in the C_4_ CCM (Figure 5; Hatch and Burnell, 1990), the conversion of atmospheric CO_2_ to HCO_3_^−^.

Transgenic approaches have been used to test the suggestion that only enough CA is present in the M cytosol of C_4_ plants to not limit photosynthesis (Hatch and Burnell, 1990). In one study (von Caemmerer et al., 2004), WT plants of the C_4_ dicot *F. bidentis* were transformed with an antisense construct directed against transcripts encoding the C_4_-associated CA3 (von Caemmerer et al., 2004, Tetu et al., 2007). A decrease in CO_2_ assimilation rates was seen only when the transformants contained less than 20% of WT CA activity, and transformants exhibiting less than 10% of WT activity had very reduced rates of photosynthesis, about 8% of WT plants, and required a high CO_2_ environment to survive. In addition, this study also showed that the hydration rate of CO_2_ was about 58 times the photosynthetic rate. Taken together, these results indicate that CA is not limiting photosynthesis in *F. bidentis*; however, the CO_2_ response curves of the transformants indicated that a cytosolic CA is essential for an efficient C_4_ CCM in this dicot species (von Caemmerer et al., 2004).

Maize plants carrying mutations in genes encoding two isoforms of CA that have been correlated with C_4_ photosynthesis (*Zm*Ca1 and *Zm*Ca2; Studer et al., 2014) demonstrated that CA is not limiting for growth in this C_4_ monocot species. Unlike the *F. bidentis* CA3 antisense plants, however, both the *ca1* single mutant and the *ca1ca2* double mutant, which contained 3% of WT maize CA activity, showed no impairment in CO_2_ assimilation at ambient levels of CO_2_. It was not until the concentration of CO_2_ was sub-ambient that a decrease in CO_2_ assimilation was detected (Studer et al., 2014). It was concluded from gas exchange and carbon isotope data, and CA and PEPC activities, that the *ca1* mutant contains only enough CA activity to supply PEPC with HCO_3_^−^, while the activity in the double mutant is below this level, and the plants rely, at least to some extent, on the uncatalyzed conversion of CO_2_ to HCO_3_^−^ (Studer et al., 2014). Clearly, while CA in both WT maize and *F. bidentis* is not rate limiting for photosynthesis, and is necessary for efficient operation of the C_4_ pathway when CO_2_ availability to the leaf is limited (Boyd et al., 2015), differences exist between these two C_4_ species with respect to the levels of CO_2_ that result in impaired CO_2_ assimilation. The basis for this discrepancy may be structural, enzymatic, or a combination of mechanisms (Studer et al., 2014, Ludwig, 2016).

### A Ubiquitous, Basal Carbon-Concentrating Mechanism in Plants

A mitochondrial βCA along with the γ and γ-like CAs associated with the mitochondrial Complex I are proposed to be part of a mechanism found in all plants that facilitates the fixation of mitochondrial respiratory CO_2_ in the chloroplasts (Figure 5; Zabaleta et al., 2012). Relative to WT plants, *Arabidopsis* mutants lacking the mitochondrial *AtβCA6* gene show a decrease in leaf area and overall biomass, inhibition of growth at low CO_2_, and a significant increase in respiration rates (Jiang et al., 2014). In contrast, the overexpression of *AtβCA6* in *Arabidopsis* resulted in larger plants with higher shoot fresh and dry weights, and decreased rates of respiration compared with WT plants (Jiang et al., 2014). Interestingly, there appeared to be no significant difference in photosynthetic rates although the CO_2_ compensation point of the knockout lines was reportedly increased relative to WT values. From this work, the authors suggested that increasing expression levels of mitochondrial *Atβ*CA6 affect cellular respiration, which impacts positively on biomass production.

### Carbonic Anhydrase Activity and Photosystem II

The finding that acetazolamide inhibited photosystem II (PS II) activity (Swader and Jacobson, 1972) raised the possibility that CA activity was associated with PSII. Later, Stemler (1997) presented evidence that included the finding of low levels of CA activity with thylakoid preparations and even core PSII fractions. The discovery of *Cr*CAH3, in the thylakoid lumen of *C. reinhardtii* (Karlsson et al., 1998), resulted in two different hypotheses as to its physiological role. In one proposal, CAH3 functions in light-driven generation of CO_2_ from accumulated HCO_3_^−^, taking advantage of the low pH of the thylakoid lumen to drive the reaction toward CO_2_ formation (Raven, 1997, Hanson et al., 2003, Moroney and Ynalvez, 2007). A competing hypothesis was that *Cr*CAH3 was required on the oxidizing side of PSII (Park et al., 1999, Villarejo et al., 2002). Since *Cr*CAH3 is an αCA, this hypothesis presented an attractive physiological role for αCAs in plants. However, evidence over the past 10–15 years strongly argues against a role for CA in PSII, with probably the most persuasive argument being the lack of CA in any of the crystal structures of PSII to date.

Most cyanobacteria do not have an αCA, and for most cyanobacteria, the only CA in the cell is in the carboxysome as part of the CCM. In *Arabidopsis*, total chloroplast (intact chloroplasts) proteome studies indicate the only CAs in the chloroplasts are *β*CA1, *β*CA2, and *β*CA5 (Friso et al., 2004; Ferro et al., 2010). The other CA reported to be in the chloroplast, αCA1, has not been detected in proteome studies to date, and no other αCA has been found in chloroplasts. None of the *Arabidopsis* β-type CAs (*Atβ*CA1, *Atβ*CA2 and *Atβ*CA5) have a leader sequence consistent with a thylakoid lumen location, and only the stromal *Atβ*CA1 is present at high levels in chloroplasts of photosynthetically active cells. Finally, Hillier et al. (2006) and McConnell et al. (2007) found no CA activity in highly active PSII preparations using the very sensitive membrane inlet mass spectrometry assay. They convincingly argued that any CA activity associated with PSII was due to contamination. This is not surprising as stromal CA activity is extremely high and even a relatively low level of contamination by this protein could result in measurable activity in enriched PSII preparations (McConnell et al., 2007).

### Stomatal Movement and Development

Increased transcript abundances are found for the genes encoding *Atβ*CA1 and *Atβ*CA4 in *Arabidopsis* guard cells (Hu et al., 2010 and references therein). While single *Atβca1* and *Atβca4* T-DNA mutants demonstrated no CO_2_-sensitive phenotype compared with WT plants, the double *ca1ca4* mutant showed impaired stomatal conductance in response to changing CO_2_ concentration as well as higher stomatal numbers and density (Hu et al., 2010). Consequently *Atβ*CA1 and *Atβ*CA4 were implicated in guard cell movement through a role in the early steps of the CO_2_ signaling pathway, and were suggested to function also in guard cell development (Hu et al., 2010). Recent work using reconstituted systems in *Xenopus* oocytes has suggested a model in which *Atβ*CA4 functions alongside the aquaporin PIP2;1 at the guard cell plasma membrane, influencing intracellular CO_2_/HCO_3_^−^ levels, which when elevated, enhance S-type anion channel activity and stomatal closure (Figure 5; Wang et al., 2016). As yet, no direct interactions between *Atβ*CA4 and PIP2;1 have been reported.

The *Arabidopsis ca1ca4* mutants show an inverted response to CO_2_ relative to WT plants in that, at high CO_2_, they have increased stomatal numbers in cotyledons and mature leaves (Engineer et al., 2014). Taking these results into account, as well as the earlier characterization of the double mutant (Hu et al., 2010), a preliminary model for the control of stomatal development has been constructed and involves an extracellular signaling pathway mediated by CA (Engineer et al., 2014, Engineer et al., 2016). Not all the components or steps in the model have been identified (Engineer et al., 2014, Engineer et al., 2016); however, it has been proposed that CA activity is necessary for the increased expression of the genes encoding the epidermal patterning factor EPF2, and the CO_2_-inducible protease that cleaves it, facilitating its binding to the receptor kinase ERECTA, which has been implicated in the regulation of stomatal development (Figure 5; Shpak, 2013).

### Biotic and Abiotic Stress Responses

Chloroplastic βCAs from C_3_ plants are part of a defense mechanism that is induced upon attack by various pathogens (Figure 5; Slaymaker et al., 2002, Restrepo et al., 2005, Jung et al., 2008, Wang et al., 2009, Collins et al., 2010). In tobacco and *Arabidopsis*, the CAs have been identified as salicylic-acid-binding proteins that function in an antioxidant role during viral infections (Slaymaker et al., 2002, Wang et al., 2009). Recombinant inbred lines of *Arabidopsis* with resistance to the insect herbivore, *Plutella xylostella*, had at least a 2-fold increase in abundance of *Atβ*CA1 and *Atβ*CA4 proteins (Collins et al., 2010).

Salinity induces an increase in βCA transcript abundance in maize, and it was suggested that this response paralleled the antioxidant role seen with the biotic stressors described above (Figure 5; Kravchik and Bernstein, 2013). Both salinity and an osmotic stress treatment using polyethylene glycol led to an increase in rice seedling total CA enzyme activity, and the level of mRNA coding for a predicted chloroplastic CA isoform (Yu et al., 2007). Overexpression of the rice CA in *Arabidopsis* led to improved growth on media containing salt compared with WT *Arabidopsis* (Yu et al., 2007).

### Amino Acid Biosynthesis

Cytosolic CAs have been implicated in affecting amino acid biosynthesis levels (Figure 5; Raven and Newman, 1994). While PEPC, the primary carboxylase of C_4_ plants, uses HCO_3_^−^ produced by cytosolic βCA activity to form C_4_ acids, as part of the C_4_ CCM, in C_3_ plants an estimated 50% of the free aspartate pool is created by PEPC activity (Melzer and O'Leary, 1987). *Arabidopsis* double knockout mutants of the *Atβca2* and *Atβca4* genes, which code for cytosolic CAs, showed reduced growth rates and chlorosis of the younger leaves relative to WT plants when grown at 200 μL L^−1^ CO_2_. This phenotype was ameliorated when the plants were grown under high levels (1000 μL L^−1^) of CO_2_ (DiMario et al., 2016). The *Atβca2ca4* double mutants also demonstrated reduced levels of aspartate, and a concomitant increase in glycine and serine levels (DiMario et al., 2016). The low CO_2_ growth phenotype and amino acid profile could be mitigated by complementation of the double mutant with the *AtβCA2* gene (DiMario et al., 2016). The elevated amounts of glycine and serine in the double mutant were unanticipated and hints CA activity affecting other biochemical pathways.

### Metabolism of Nitrogen-Fixing Root Nodules

The nitrogen-fixing root nodules of numerous legumes contain relatively high CA activity (Atkins, 1974), and transcripts encoding βCAs have been isolated from the nodules of several species (Coba de la Peña et al., 1997, Kavroulakis et al., 2000, Flemetakis et al., 2003). The location of these transcripts and the proteins they encode changes during maturation of the nodules, and this suggests that the role of the enzymes likely varies over the course of nodule development (Kavroulakis et al., 2000, Flemetakis et al., 2003). The functions put forward involve the provision of HCO_3_^−^ for processes such as amino acid and lipid biosynthesis and gluconeogenesis in the early developmental stages, and the release of CO_2_ generated from bacteroid respiration to the rhizosphere in mature nodules (Figure 5; Kavroulakis et al., 2000, Flemetakis et al., 2003). However, these have not been supported experimentally. The presence of additional forms of CA in nitrogen-fixing nodules complicates the identification of the precise role(s) of the βCA enzymes (Gálvez et al., 2000, Flemetakis et al., 2003, Yahyaoui et al., 2004; supplemental data; Kalloniati et al., 2009, Tsikou et al., 2011, Tang et al., 2014).

### Lipid Biosynthesis

Fatty acid synthesis is a primary metabolic pathway in which acetyl-CoA carboxylase (ACC) uses HCO_3_^−^ to carboxylate acetyl-CoA to produce malonyl-CoA, the building block of fatty acid chains (Sasaki and Nagano, 2004). In plants, the production of acyl chains takes place in the chloroplast while their utilization occurs in essentially every cellular compartment (Ohlrogge and Jaworski, 1997). Since ACC requires HCO_3_^−^, and previous results have shown significant expression and activity of CA in cotton seedlings (Hoang et al., 1999, Hoang and Chapman, 2002a), Hoang and Chapman (2002b) examined the level of radiolabeled acetate incorporation into lipids in cottonseed embryos and tobacco cell suspensions. When embryos and suspension culture cells were incubated with [^14^C]acetate in the presence of the CA inhibitor, ethoxyzolamide, the rates of lipid synthesis were greatly decreased. Antisense tobacco lines with 5% of WT CA activity (Price et al., 1994) also showed lower levels of radiolabeled lipids (Hoang and Chapman, 2002b), which is consistent with the transgenic plants demonstrating reduced rates of C_i_ entering the chloroplast (Price et al., 1994). Hoang and Chapman (2002b) suggested that CA activity traps C_i_ within chloroplasts in the form of HCO_3_^−^, which is then used by ACC in fatty acid synthesis (Figure 5).

## Conclusions and Perspectives

Plants have many genes encoding α-, β- and γ-type CAs, which are found in most tissues and many intracellular compartments. In addition, alternative splicing and multiple transcription start sites have been shown in a number of βCAs, often leading to different proteins targeted to different organelles. Programs developed to predict protein targeting should be used with caution, particularly when working with monocot CAs or CAs predicted from gene models. The number of CA genes is relatively similar in monocot and dicots, and in plants using C_3_ or C_4_ photosynthesis, or CAM. Evidence is building that, during the evolution of the C_4_ pathway, C_3_ genes coding for CA were co-opted through changes in *cis*-regulatory sequences, modification of posttranscriptional controls, and/or recruitment of different transcription factors. CAs, while clearly important in photosynthesis, are also required for other metabolic pathways as well as signaling and developmental pathways.

## Funding

This work was supported by the University of Illinois as part of the Bill & Melinda Gates Foundation-funded Realizing Increased Photosynthetic Efficiency (RIPE) consortium, by NSF grant IOS-1146597 to J.V.M. and the Australian Research Council Discovery Projects DP130102243 and DP150101037 to M.L.

## Author Contributions

R.J.D., H.C., A.M., M.L., and J.V.M. all contributed to the writing of the original draft and to the reviewing and editing of the manuscript.

## Figures and Tables

**Table 1:** Total Number of α, β, and γ Carbonic Anhydrase Genes in Different Plants.

Plant type	Species	PS type	Type and number of CA genes		
			α	β	γ
Moss	*Physcomitella patens*	C_3_	5	6	5
Club moss	*Selaginella moellendorffii*	C_3_	10	5	4
Dicots	*Arabidopsis thaliana*	C_3_	8	6	5
	*Medicago truncatula*	C_3_	8	7	4
	*Vitis vinifera*	C_3_	5	6	3
	*Populus trichocarpa*	C_3_	8	7	5
Monocots	*Brachypodium distachyon*	C_3_	6	4	3
	*Oryza sativa*	C_3_	9	3	4
	*Setaria italica*	C_4_	9	4	3
	*Sorghum bicolor*	C_4_	9	5	3
	*Ananas comosus*	CAM	4	3	3

Genes were identified based on multiple sequence alignment in Clustal Omega using *Arabidopsis* carbonic anhydrase (CA) genes as query. Sequences for *Physcomitrella patens*, *Selaginella moellendorffii*, *Medicago truncatula*, *Vitis vinifera*, *Populus trichocarpa*, *Brachypodium distachyon*, *Oryza sativa*, *Setaria italica*, *Sorghum bicolor* were obtained from Phytozome (https://phytozome.jgi.doe.gov) and NCBI. Sequences for *Ananas comosus* were obtained from CoGe as described in Ming et al., 2015. γCA gene numbers include γ-like CA genes.

PS, photosynthetic type; C_3_, C_3_ photosynthesis; C_4_, C_4_ photosynthesis; CAM, crassulacean acid metabolism.

**Table 2:** Experimentally Derived Subcellular Locations of Plant β Carbonic Anhydrase Isoforms.

Gene/Accession No.	Protein	Location	Reference
**MONOCOT**			

*Neurachne alopecuroidea* (C_3_)			
NaloCA1a	βCA1a	Chloroplast	Clayton et al. (2016)
NaloCA1b	βCA1b	Cytosol	Clayton et al. (2016)
*Neurachne munroi* (C_4_)			
NmunCA1a	βCA1a	Cytosol	Clayton et al. (2016)
NmunCA1b	βCA1b	Cytosol	Clayton et al. (2016)
NmunCA2a	βCA2a	Cytosol	Clayton et al. (2016)
NmunCA2b	βCA2b	Chloroplast	Clayton et al. (2016)

**DICOT**			

*Arabidopsis thaliana* (C_3_)			
AT3G01500	βCA1	Chloroplast	Fabre et al. (2007)
			Hu et al. (2015)
AT5G14740	βCA2	Cytosol	Fabre et al. (2007)
			DiMario et al. (2016)
AT1G23730	βCA3	Cytosol	Fabre et al. (2007)
AT1G70410	βCA4.1	Plasma membrane	Fabre et al. (2007)
			Hu et al. (2010)
			Hu et al. (2015)
			Wang et al. (2014)
			DiMario et al. (2016)
AT1G70410	βCA4.2	Cytosol	DiMario et al. (2016)
AT4G33580	βCA5	Chloroplast	Fabre et al. (2007)
AT1G58180	βCA6	Mitochondrion	Fabre et al. (2007)
			Jiang et al. (2014)
*Flaveria bidentis* (C_4_)			
AAA86939.2	βCA1	Chloroplast	Tetu et al. (2007)
AAO17573.1	βCA2	−	Tetu et al. (2007)
AAO17574.1	βCA3	−	Tetu et al. (2007)
*Flaveria pringlei* (C_3_)			
AAA86992.1	βCA1	Chloroplast	Tanz et al. (2009)
ABC41657.1	βCA2	−	Tanz et al. (2009)
ABC41658.1	βCA3	Chloroplast	Tanz et al. (2009)

Data for *Flaveria* βCA subcellular locations were obtained using chloroplast import assays, and *Arabidopsis* and *Neurachne* βCA subcellular locations were determined using fluorescent protein fusion constructs. Dash indicates that the protein is not targeted to the chloroplast. *Arabidopsis* βCA sequences are available in TAIR, *Flaveria* sequences are identified by GenBank accession numbers, and *Neurachne* sequences are as described in Clayton et al., 2016.

## Supplemental Information

Table S1. Predicted Subcellular Locations of β Carbonic Anhydrases in Various Species Using Four Protein Subcellular Location Prediction Programs: Predotar, ChloroP, TargetP, and MultiLoc, Related to Table 2
