# Supplementary material for: Plant Carbonic Anhydrases: Structures, Locations, Evolution, and Physiological Roles
Source: Mol Plant. 2017 Jan 9;10(1):30–46. doi: 10.1016/j.molp.2016.09.001 (PMC5226100; doi:10.1016/j.molp.2016.09.001)
Supplement: Table S1. Predicted Subcellular Locations of β Carbonic Anhydrases in Various Species Using Four Protein Subcellular Location Prediction Programs: Predotar, ChloroP, TargetP, and MultiLoc, Related to Table 2 [file mmc1.docx]

Table S1 Related to Table 2. Predicted subcellular locations of beta carbonic anhydrases in various species using four protein subcellular location prediction programs: Predotar, ChloroP, TargetP, and MultiLoc. Dash indicates no targeting predicted. All putative polypeptide sequences were obtained from Phytozome v11.0 genome data with the exception of Arabidopsis ([DiMario et al., 2016](#_ENREF_4)), *Flaveria* ([Tanz et al., 2009](#_ENREF_23); [Tetu et al., 2007](#_ENREF_24)), and *Neurachne* (Clayton et al., 2016) species.

| **Protein** | **Predotar^1^** | **ChloroP^2^** | **TargetP^3^** | **MultiLoc^4^** |
| --- | --- | --- | --- | --- |
| ***Physcomitrella patens* (C_3_)** |  |  |  |  |
| Pp3c24_9430V3.1 | - | Chloroplast | Chloroplast | Chloroplast |
| Pp3c7_14450V3.1 | Chloroplast | Chloroplast | Chloroplast | Chloroplast |
| Pp3c1_19190V3.2 | - | - | Other | Peroxisome |
| Pp3c20_18640V3.2 | Chloroplast | Chloroplast | Chloroplast | Mitochondrion |
| Pp3c2_15140V3.3 | - | - | Other | Cytosol |
| Pp3c16_19250V3.3 | - | - | Other | Cytosol |
| ***Selaginella moellendorffii* (C_3_)** |  |  |  |  |
| e_gw1.17.179.1 | - | - | - | Cytosol |
| gw1.4.392.1 | - | - | - | Cytosol |
| e_gw1.8.1438.1 | - | - | - | Peroxisome |
| e_gw1.4.398.1 | - | - | - | Peroxisome |
| e_gw1.4.403.1 | Chloroplast | Chloroplast | Mitochondrion | Peroxisome |
| e_gw1.26.167.1 | - | - | - | Cytosol |
| **ANGIOSPERMS** |  |  |  |  |
| **MONOCOTS** |  |  |  |  |
| ***Brachypodium distachyon* (C_3_)** |  |  |  |  |
| Bradi4g32480 | Chloroplast | Chloroplast | Chloroplast | Chloroplast |
| Bradi2g44856 | - | - | Other | Chloroplast |
| Bradi2g44870 | - | - | Chloroplast | Mitochondrion |
| ***Neurachne alopecuroidea* (C_3_)** |  |  |  |  |
| NaloCA1a | Chloroplast | Chloroplast | Chloroplast | Chloroplast |
| NaloCA1b | - | - | Other | Cytosol |
| NaloCA2a | - | Chloroplast | Chloroplast | Chloroplast |
| NaloCA2b | Chloroplast | Chloroplast | Chloroplast | Mitochondrion |
| ***Neurachne munroi* (C_4_)** |  |  |  |  |
| NmunCA1a | Chloroplast | Chloroplast | Chloroplast | Chloroplast |
| NmunCA1b | - | - | Chloroplast | Cytosol |
| NmunCA2a | Mitochondrion | - | Mitochondrion | Nucleus |
| NmunCA2b | Chloroplast | Chloroplast | Chloroplast | Vacuole |
| ***Oryza sativa* (C_3_)** |  |  |  |  |
| Os01g45274.1 | Chloroplast | Chloroplast | Chloroplast | Chloroplast |
| Os09g28910.1 | Mitochondrion | - | Mitochondrion | Mitochondrion |
| ***Setaria italica* (C_4_)** |  |  |  |  |
| Seita.2G235500.1 | Mitochondrion | - | Mitochondrion | Mitochondrion |
| Seita.5G240000.1 | - | - | Chloroplast | Chloroplast |
| Seita.5G240100.1 | - | - | Mitochondrion | Mitochondrion |
| Seita.5G240200.1 | - | - | Other | Mitochondrion |
| ***Sorghum bicolor* (C_4_)** |  |  |  |  |
| Sobic.003G234600.1 | - | - | Other | Peroxisome |
| Sobic.003G234200.6 | - | - | Other | Peroxisome |
| Sobic.003G234400.4 | - | - | Other | Cytosol |
| Sobic.003G234500.1 | - | - | Other | Cytosol |
| Sobic.002G230100.5 | Chloroplast | Chloroplast | Chloroplast | Nucleus |
| **EUDICOTS** |  |  |  |  |
| ***Arabidopsis thaliana* (C_3_)** |  |  |  |  |
| At3g01500.2 | Chloroplast | Chloroplast | Chloroplast | Chloroplast |
| At5g14740.1 | Chloroplast | Chloroplast | Other | Chloroplast |
| At1g23730.1 | Cytosol | - | Other | Cytosol |
| At1g70410.2 | - | - | Other | Peroxisome |
| At4g33580.2 | - | Chloroplast | Chloroplast | Mitochondrion |
| At1g58180.2 | Mitochondrion | - | Mitochondrion | Cytosol |
| ***Flaveria bidentis* (C_4_)** |  |  |  |  |
| AAA86939.2 | Chloroplast | Chloroplast | Chloroplast | Chloroplast |
| AAO17573.1 | - | - | Other | Cytosol |
| AAO17574.1 | - | - | Other | Peroxisome |
| ***Flaveria pringlei* (C_3_)** |  |  |  |  |
| AAA86992.1 | Chloroplast | Chloroplast | Chloroplast | Chloroplast |
| ABC41657.1 | - | - | Other | Cytosol |
| ABC41658.1 | Chloroplast | Chloroplast | Chloroplast | Chloroplast |
| ***Manihot esculenta* (C_3_-C_4_)** |  |  |  |  |
| Manes.07G038800.1 | Chloroplast | Chloroplast | Chloroplast | Chloroplast |
| Manes.10G099600.1 | Chloroplast | - | Chloroplast | Vacuole |
| Manes.18G059500.1 | - | Chloroplast | Chloroplast | Cytosol |
| Manes.15G167500.1 | Chloroplast | Chloroplast | Chloroplast | Chloroplast |
| Manes.13G029900.1 | - | - | Other | Cytosol |
| Manes.12G028400.1 | - | - | Other | Cytosol |
| Manes.17G116800.1 | Chloroplast | Chloroplast | Chloroplast | Chloroplast |
| Manes.14G066100.1 | - | - | Other | Cytosol |
| ***Medicago truncatula* (C_3_)** |  |  |  |  |
| Medtr2g009500.1 | Chloroplast | Chloroplast | Chloroplast | Cytosol |
| Medtr3g077930.1 | - | - | Other | Peroxisome |
| Medtr5g066060.3 | Mitochondrion | - | Mitochondrion | Chloroplast |
| Medtr6g034285.1 | - | - | Other | Golgi |
| Medtr3g077940.5 | - | - | Other | Cytosol |
| Medtr5g034250.2 | Mitochondria | - | Secretory | Peroxisome |
| Medtr8g064630.1 | - | - | Other | Peroxisome |
| Medtr6g006990.6 | Chloroplast | Chloroplast | Chloroplast | Chloroplast |
| ***Populus trichocarpa* (C_3_)** |  |  |  |  |
| Potri.005G156600.2 | - | - | Other | Cytosol |
| Potri.017G044700.3 | - | - | Other | Cytosol |
| Potri.007G114600.2 | - | - | Other | Chloroplast |
| Potri.005G156600.3 | - | - | Other | Cytosol |
| Potri.010G041100.4 | - | - | Other | Peroxisome |
| Potri.008G189800.1 | - | - | Other | Peroxisome |
| Potri.001G348900.13 | - | - | Other | Peroxisome |
| Potri.015G075900.1 | - | - | Other | Cytosol |
| Potri.015G076000.2 | - | - | Other | Cytosol |
| ***Vitis vinifera* (C_3_)** |  |  |  |  |
| GSVIVT01015633001 | Chloroplast | Chloroplast | Chloroplast | Chloroplast |
| GSVIVT01009165001 | Mitochondrion | Chloroplast | Chloroplast | Chloroplast |
| GSVIVT01013736001 | - | - | - | Peroxisome |
| GSVIVT01032466001 | Chloroplast | Chloroplast | Chloroplast | Chloroplast |
| GSVIVT01007745001 | - | - | - | Cytosol |
| GSVIVT01007742001 | - | - | - | Cytosol |
| GSVIVT01032226001 | - | - | Other | Nucleus |
| GSVIVT01007741001 | ER | Chloroplast | Chloroplast | Extracellular |

Small *et al.*, 2004^1^; Emanuelsson *et al.*, 1999^2^; Emanuelsson *et al.*, 2007^3^; Höglund *et al.*, 2006^4^
